# An exploratory study of knowledge, attitudes, and beliefs related to tobacco use and secondhand smoke among women in Aleta Wondo, Ethiopia

**DOI:** 10.1186/s12905-018-0640-y

**Published:** 2018-09-24

**Authors:** Anne Berit Petersen, Lisa M. Thompson, Gezahegn Bekele Dadi, Alemu Tolcha, Janine K. Cataldo

**Affiliations:** 1 School of Nursing, Loma Linda University, West Hall, 11262 Campus Street, Loma Linda, CA 92350 USA; 2 Nell Hodgson Woodruff School of Nursing, Emory University, 1520 Clifton Road, Suite 226, Atlanta, GA 30322 USA; 3 School of Nursing and Midwifery, College of Medicine and Health Sciences, Hawassa University, PO Box 1560, Hawassa, Ethiopia; 4 School of Public and Environmental Health, College of Medicine and Health Sciences, Hawassa University, PO Box 1560, Hawassa, Ethiopia; 5 Department of Physiological Nursing and Center for Tobacco Control Research and Education, University of California, San Francisco, 2 Koret Way, N611Q, San Francisco, CA 94143 USA

**Keywords:** Tobacco use, Secondhand smoke exposure, Ethiopia, Women, Knowledge, Attitudes and beliefs, Tobacco messaging, Khat use, Religion

## Abstract

**Background:**

By 2030, the Sub-Saharan African region is projected to be the epicenter of the tobacco epidemic. While smoking prevalence is currently low among women (< 2%), the prevalence among men (7.7% overall and up to 27% depending on region) makes exposure to secondhand smoke a pressing concern for women and children. To prevent the uptake of smoking among women and address tobacco-related risks, including secondhand smoke exposure, a greater understanding of women’s related perceptions is needed. The purpose of this study was to explore Ethiopian women’s knowledge, attitudes, and beliefs related to tobacco use and secondhand smoke exposure, and the potential influence of contextual factors including; khat use, exposure to pro- and anti-tobacco messaging, and religious affiliation.

**Methods:**

A cross-sectional study using a systematic household sampling technique and an adapted interviewer-administered survey was conducted in Southern Ethiopia. The survey was administered to 353 women, 18–55 years of age, in Aleta Wondo town and surrounding districts between August–October 2014 (95.2% cooperation rate).

**Results:**

General awareness of harm associated with personal tobacco use and exposure to secondhand smoke was high (> 94%); however, specific knowledge of associated health-risks was limited. More than 96% perceived female tobacco use as socially unacceptable. At the same time, more than 70% were able to name potential benefits of using tobacco for both personal consumption and non-personal use. Respondents reported greater experimentation with *khat* versus tobacco and 73% reported that their religion significantly influenced their tobacco-related attitudes. Overall, there were higher reports of exposure to anti-tobacco (70%) versus pro-tobacco (49%) messaging, in the last 30 days.

**Conclusions:**

The high level of awareness of health risks associated with tobacco use and SHS exposure and the high exposure to anti-tobacco messaging are community-level strengths that can proactively be built on to prevent the projected disease burden associated with tobacco. Findings have implications for the development of contextualized gender-specific tobacco control interventions, particularly in relation to the promotion of smoke-free homes.

## Background

Globally, tobacco is widely recognized as one of the leading threats to population health, accounting for more than 6 million deaths per year [1, 2]. Without urgent interventions, by 2030 the death toll from tobacco is projected to reach 8 million people per year [1]. Of particular concern is that 80% of these deaths are projected to occur in low and middle-income countries (LMICs), and by 2030 sub-Saharan Africa is projected to be the next epicenter of this epidemic [2, 3]. While most LMICs are in the early stages of a tobacco epidemic, evidence suggests that the epidemic may unfold differently across LMICs than has previously been observed in high-income settings, particularly among women [4–8].

Active tobacco smoking has been causally linked to an increased risk of acquiring and dying from numerous diseases, including many types of cancers, cardiovascular disease, chronic obstructive pulmonary disease, and diabetes [9]. Women who smoke are essentially at the same risk for these diseases as men and face an increased risk for gender-specific diseases and health threats such as cervical cancer, breast cancer, osteoporosis, infertility, dysmenorrhea, premature menopause and adverse birth outcomes including spontaneous abortions, stillbirths, and low infant birth weight [1, 10]. In addition, SHS exposure has been causally linked to a range of chronic diseases and health problems for both women and children, including increased risk of lung cancer and cardiovascular disease for women and increased risk of sudden infant death syndrome, delays in physical development and cancer for children [1, 9].

Due to the unique gender-specific risks associated with tobacco use, there is a need for tailored interventions that support women’s efforts to advocate for their own health and that of their family members. Article 4 of the World Health Organization Framework Convention on Tobacco Control (WHO FCTC) states that every person should be informed of the health consequences of tobacco, including its addictive nature and the risks associated with SHS exposure [11]. However, in order to inform gender-sensitive tobacco control policies and interventions greater understanding of the knowledge, attitudes, and beliefs (KABs) related to tobacco use and SHS exposure among women in LMICs is needed.

Ethiopia is one of the most recent countries to adopt the WHO FCTC and pass comprehensive tobacco control legislation [12, 13]. However, the available data on tobacco use and tobacco-related KABs needed to inform contextualized public health interventions is minimal [14]. To date, the investigations have primarily been conducted in urban settings (although 80% of the population lives in rural areas), and among special populations such as adolescents, secondary students, university students, and instructors [14–20].

Based on the 2011 Ethiopian Demographic Health Survey (EDHS) the overall prevalence of female smoking is low (< 2%); however, the prevalence among males is 7.7% and varies greatly depending on region (up to 27%) [14]. There is also evidence to suggest that these rates are increasing. A study by Reda et al., conducted in a rural setting in southeastern Ethiopia, reported a 28.8% overall current smoking rate with male smoking as high as 38.6% [21]. This study is also the only community-level investigation that has explored KABs related to adult tobacco use in Ethiopia. Seventy-three percent of the respondents (*n* = 548; 75% male) indicated that they believed smoking tobacco was associated with harmful health effects such as heart disease, respiratory problems, and lung cancer. Respondents’ beliefs regarding SHS risks were not explored, yet in 52% of the households surveyed, smoking was allowed indoors, and in 33% of households, indoor smoking occurred daily [21]. The single study by Reda et al. provides a limited understanding of the perceived risks associated with tobacco use among women at the community level in Ethiopia; illuminating the gap in the literature related to underlying tobacco-related KABs [21]. Furthermore, in previous studies conducted among women in LMICs, urbanicity, or differing degrees of interaction and identification with urban settings has been found to be predictive of more favorable attitudes toward smoking [22–24]. *Khat* (*Catha edulis;* a plant-based alkaloid stimulant native to the Horn of Africa) is legal and widely used in Ethiopia, and has been posited as a potential gateway to tobacco in other settings in Africa [25, 26]. Therefore, the purpose of the present study was to explore rural Ethiopian women’s KABs related to maternal tobacco use and SHS exposure, and to describe the prevalence of a number of contextual factors that, based on the literature, have the potential to influence these KABs, including; urbanicity, *khat* use, current exposure to pro- and anti-tobacco messaging, and the influence of religion on tobacco-related attitudes.

## Methods

### Sample

Between August and October 2014 we conducted a cross-sectional household-level study in four *kebeles* (smallest administrative units); two within the rural town of Aleta Wondo, Ethiopia and two in the immediate outlying rural area. This study was part of a more extensive study which aimed to explore maternal and child exposure to both secondhand tobacco smoke and cooking fire smoke and related KABs, for which the inclusion criteria included: (a) female 18 to 55 years of age, (b) continuous residence in Ethiopia for the past 5 years, (c) currently have children or grandchildren (≤12 years) living in the household, (d) being a primary cook in the household, and (e) fluent in either the Amharic and/or the Sidama dialects. Because of the nascent nature of tobacco control research in Ethiopia, this is a descriptive exploratory study and represents first steps at identifying attitudes, knowledge (harm perceptions) and behaviors of women related to smoking in rural Ethiopia.

Based on the literature review conducted on studies evaluating tobacco-related KABs among women in LMICs, a sample size of 400 was deemed appropriate for this descriptive study. When constructing confidence intervals around the sample proportions a minimum sample size of 377 would be needed to limit the margin of error to 5% (http://www.raosoft.com/samplesize.html). The proposed sample size took into account incomplete surveys and provided adequate power (> 80%) to detect an odds ratio of 1.8 or higher to be statistically significant [27].

The recruitment procedures and sampling technique employed in this study have been extensively described elsewhere [28]. In brief, participants were recruited using a systematic household-level sampling technique [29]. While all four *kebeles* included in the sampling frame are classified as rural in the *Demographic Health Surveys* [30], the influence of varying degrees of urbanicity was integrated into the study design by selecting two *kebeles* located in the town and two in the surrounding rural area. Based on available local census reports, each of the four *kebeles* was comprised of approximately 1600 households, and 25% of the sample was drawn from each *kebele*. Trained interviewers started in the geographical center of the *kebele*. Randomized numbers (1 to 10) were used in the selection of the first house, after which, every third house was selected radiating out in four different directions in the *kebele*. Using a script, interviewers invited one eligible woman in the home to participate. If she refused or no one was eligible or available, the interviewer approached the next immediate house. Written consent was obtained, and all study procedures were reviewed and approved by the human subjects review boards of the University of California-San Francisco, San Francisco, CA, U.S.A and Hawassa University, Hawassa, Ethiopia.

### Instrument development and variables

An interviewer administered structured questionnaire developed by Bloch et al. [31], was adapted for use in this study [32]. This instrument assessed the following main outcome variables: (a) knowledge of maternal health risks related to cigarettes, other tobacco products, SHS, and addiction; (b) perceived acceptability of women’s tobacco and *khat* use; (c) influence of religious affiliation on tobacco-related attitudes, (d) perceived benefits of cigarettes and smokeless tobacco; (e) perceived benefits of non-personal uses of tobacco; (f) exposure to anti-tobacco information; and (g) exposure to cigarette advertising. The Global Multidimensional Poverty Index (MPI) was used to describe the socioeconomic status of the sample. The MPI is an acute measure of poverty that takes into consideration multiple sources of deprivation experienced simultaneously by a household. “Multidimensional poverty” is defined as deprivation in 33.3% or more of 10 indicators [33].

Detailed descriptions of the measures, and translation and adaptation processes have been presented elsewhere [28]. We used World Health Organization guidelines for translation and adaptation of instruments and recommendations from the International Agency for Research on Cancer [34, 35]. The questionnaire was translated into the Sidama and Amharic dialects by language experts, reviewed by expert bilingual panels, back-translated, and pre-tested in the Sidama dialect with two focus groups. Discrepancies at each step were addressed in an iterative manner with members of the expert panels until consensus on final version was reached.

### Data analysis

The data were described using frequency and percent of total for categorical variables, and mean and standard deviation for continuous variables. Six items addressing tobacco-related harm perceptions (Do you think a woman who smokes cigarettes [uses smokeless tobacco products] harms her own health or not?; If a mother smokes cigarettes [uses smokeless tobacco products] during her pregnancy, do you think her smoking can harm her unborn baby’s health or not?; Once someone has started smoking cigarettes, do you think it would be difficult to quit or not?; Do you think the smoke from other people’s cigarettes is harmful or not?) were combined to create a composite harm perception variable (% correct). Relationships between covariates theorized to be associated with knowledge and harm perceptions were explored using Independent *t*-tests and Chi-square or Fisher’s Exact Tests as appropriate. Significance is reported at an alpha level of 0.05. All statistical analyses were performed using SPSS Version 22 [36].

## Results

### Sample characteristics

Out of 708 households contacted, 137 were not at home, and 369 were eligible. Among the eligible women approached, 16 declined, and 353 completed the survey, resulting in a 95.2% cooperation rate. The majority of surveys were conducted in the Sidama dialect (78.2%) and the remaining in Amharic, with a median administration time of 21 min. A majority of respondents were married (94.9%), of Sidama descent (71.5%), Protestant (79.2%), more than 50% reported having completed five years or less of formal education, and 99.7% reported relying on biomass fuel for cooking (Table 1). Among the households classified as “multidimensionally poor” (13.6%, *n* = 48 households), the intensity of deprivation among the individuals in these households (*n* = 271 household members) ranged from 33.3 to 94.7%, and the average intensity of deprivation was 0.74 (i.e., deprived in 74% of the indicators).

**Table 1:** Sample characteristics

*N* = 353	*Mean*	SD
Variable		
Maternal age (years), mean, ± SD^a^	29.4	± 6.9
Education (years), mean, ± SD	5.7	± 3.8
	*n*	(%)
Education		
None, number (%)	46	(13.0)
1 to 5 years, number (%)	136	(38.5)
6 to 10 years, number (%)	136	(38.5)
11 or more years, number (%)	35	(9.9)
Marital status		
Married	334	(94.9)
Other	18	(5.1)
Currently pregnant	31	(8.8)
Ethnicity (tribal association)		
Sidama	251	(71.5)
Amhara	58	(16.5)
Gurage	20	(5.7)
Oromo	14	(4.0)
Other	8	(2.3)
Religious affiliation (% yes)	351	(100)
Religious affiliation, specified		
Protestant	278	(79.2)
Orthodox Christian	61	(17.4)
Muslim	10	(2.8)
Catholic	2	(0.6)
Municipality		
Rural	179	(50.7)
Semi-urban/town	174	(49.3)
Multidimensional Poverty Index (% MPI poor^b^)	48	(13.6)
Individual MPI Indicators:^c^		
No one in household ≥5 years schooling	38	(11.3)
School-age child not enrolled in years 1–8	31	(8.8)
Household has experienced child (<5 years) death	107	(30.3)
No electricity	71	(20.1)
No access to clean drinking water or > 30 minute walk	85	(24.1)
No toilet or improved latrine or toilet is shared	64	(18.1)
Dirt, sand or dung flooring	150	(42.5)
Cooks with biomass fuel (wood, charcoal or dung)	352	(99.7)
Household does not own more than one of following: Radio, TV, telephone, bike, or motorbike, and do not own a car or tractor	244	(69.1)

^a^Six participants gave “Don’t know” response but indicated they were less than 55 years of age

^b^Percent of sample defined as “multidimensionally poor” (i.e. Household is deprived in some combination of indicators whose weighted sum exceeds 30% of all deprivations) (Alkire & Santos, 2010, p. 2)

^c^Number and percent (%) deprived within each individual indicator

None of the respondents reported current use of either cigarettes or smokeless tobacco products, and less than 1% reported having ever tried cigarettes (0.8%; *n* = 3) and smokeless tobacco products (0.3%; *n* = 2). In contrast, 14.4% (*n* = 50) reported that smoking occurred daily inside their homes. Also, 11% of respondents (*n* = 39) reported having ever chewed *khat*, with 3.4% (*n* = 12) reporting having chewed within the last 30 days. A significant difference in ever *khat* use was observed between respondents residing in the town (20.1% (*n* = 35) compared with respondents residing in an outlying rural *kebele* (2.2%, *n* = 4, *p* < .001).

### Harm perceptions associated with tobacco use and secondhand smoke exposure

Respondents perceptions of harm associated with cigarettes, smokeless tobacco, and SHS exposure are presented in Table 2. Overall awareness of personal health risks associated with tobacco use and SHS exposure was high (> 94%); however, respondents’ knowledge about specific health effects or diseases caused by these substances was limited. While nearly all respondents (98.0%, *n* = 346) believed that a woman who smokes cigarettes harms her own health, unspecified reference to “lung disease” was the most frequently identified health effect or disease caused by cigarette smoking (70.5%, *n* = 249), followed by other respiratory and breathing problem symptoms (e.g., coughing) (Table 2). Far fewer respondents (< 15%) named specific diseases such as lung cancer and heart disease, and approximately 10% of the respondents were unable to name one specific health effect or disease caused by smoking cigarettes (i.e., responded with “don’t know” (*n* = 34)).

**Table 2:** Knowledge, attitudes, and beliefs related to tobacco and khat use

*N* = 353		
Variable	*n*	%
Cigarette use		
Think it is acceptable for women to smoke (% “yes”, “it depends”, “don’t know”)	9	(2.6)
Smokeless tobacco product use		
Think it is acceptable for women to use (% “yes”, “it depends”, “don’t know”)	7	(2.1)
*Khat* use		
Think it is acceptable for women to chew *khat* (% “yes”, “it depends”, “don’t know”)	12	(3.4)
Cigarette harm perceptions		
Think cigarette smoking harms woman’s health (% yes)	346	(98.0)
*Specific diseases/conditions named:*^a^		
Lung disease (unspecified)	249	(70.5)
Coughing	102	(28.9)
Respiratory/breathing problems	76	(21.5)
Cancer – lung	51	(14.4)
Addiction	48	(13.6)
Heart disease	48	(13.6)
Don’t know	34	(9.6)
Think cigarette smoking during pregnancy harms unborn baby (% yes)	334	(94.6)
*Specific diseases/conditions named:*^a^		
Lower birth weight	129	(36.5)
Lung disease/Respiratory problem - unspecified	67	(19.0)
Abnormal/ “sickly” baby – unspecified	14	(4.0)
Don’t know	128	(36.3)
Think secondhand smoke is harmful (% yes)	344	(97.5)
*Specific diseases/conditions named:*^a^		
Lung disease – unspecified	182	(51.6)
Respiratory/breathing problems	70	(19.8)
Headache/dizziness	29	(8.2)
Heart disease	29	(8.2)
Lung cancer	16	(4.5)
Nausea/vomiting	15	(4.2)
Don’t know	86	(24.4)
Smokeless tobacco harm perceptions		
Think a woman who uses smokeless tobacco harms her health (% yes)	331	(93.8)
Think a mother who uses smokeless tobacco harms her unborn baby (% yes)	333	(94.6)
Perceptions related to addiction		
Think once someone started smoking cigarettes it would be difficult to quit (% yes)	283	(80.2)
Perceptions related to tobacco use and religion		
How much influence does your religion have on your attitude toward tobacco use?^b^		
“A lot”	257	(73.2)
“Somewhat influential”, “A little”, or “Not at all”	94	(34.0)
Perceive tobacco as ‘bad’ (% yes)^c, d^	190	(100)
Perceive that one’s religion views tobacco as ‘bad’ (% yes)^c, e^	190	(100)

^a^May be greater than 100% because participants were permitted to name more than one disease/condition

^b^Question: *“How much influence would you say that your religion influences your attitude toward tobacco use?”*

^c^Follow-up question was of a subset (*n* = 190) of total sample

^d^Question: *“Do you think that tobacco is bad?”*

^e^Question: *“Does your religion say that tobacco is bad?”*

Findings were similar when respondents were queried about perceptions of harm concerning smoking and use of smokeless tobacco products on an unborn baby’s health (Table 2). While 36.5% (*n* = 129) identified “lower birth weight” as a health effect caused by maternal smoking, other responses tended to refer to nonspecific respiratory symptoms or descriptions of “abnormal” or “sickly” babies. However, 36.3% (*n* = 128), reported that they were unable to name a specific health effect or disease affecting unborn babies as a result of maternal smoking (i.e., responded “don’t know”). Similarly, nearly all respondents (97.5%, *n* = 344) reported that they believed SHS was harmful to health. However, nearly one quarter (*n* = 86) of the respondents were unable to name a specific health effect or disease caused by SHS.

**Fig. 1:**
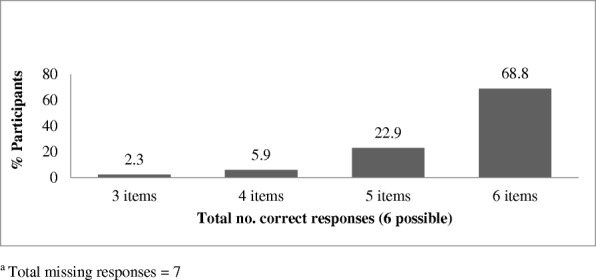
Sample level frequencies of correct responses (% yes) to six tobacco-related harm perception items (*N* = 353)^a^

Among the six items which aimed to explore respondents perceptions of harm related to cigarettes and smokeless tobacco products, understanding of addiction in relation to cigarette smoking was the most limited, with 18.5% (*n* = 65) indicating that they did not “think it would be difficult to quit once someone had started smoking cigarettes.” The sample level frequencies of correct responses (% yes) to the six tobacco-related harm perception items are presented in Fig. 1. However, no significant differences were observed between the tobacco harm perception composite variable and the following covariates; age, years of education, poverty (MPI rate), place of residence, ethnicity, religious affiliation, reported daily occurrence of smoking in the home, and exposure to pro- and anti-tobacco messaging.

### Attitudes and beliefs related to tobacco and khat use

Less than 3% of respondents believed that female tobacco use was acceptable (Table 2). Concerning *khat* use, 3.4% reported that it was “acceptable for women” to use *khat* or that “it depends”, or that they “don’t know”*.* When respondents gave an “it depends” response they were asked, “what does it depend on?”. These responses included: a “woman’s age”, “location where *khat* is being chewed”, “her religion”, “her culture” and “[characteristics of] her own community.” There were no significant differences between respondents in regards to their attitudes toward female tobacco or *khat* use based on place of residence.

Seventy-three percent (*n* = 257) of respondents reported that their religion had “a lot” of influence on their attitudes toward tobacco (Table 2), however, no significant differences were found in response to this item based on specific religious affiliation or place of residence. When a subsample (*n* = 190) was queried about whether they perceived tobacco to be “bad”, all respondents responded “yes”. The response was identical when asked, “Does your religion say tobacco is bad?”

### Perceived benefits and motivations for personal and non-personal uses of tobacco products

When asked to provide reasons why someone might start using cigarettes the most common response was “don’t know” (26.1%, *n* = 92), followed by stress (22.9%, *n* = 81), experimentation (21.8%, *n* = 77), and peer pressure (16.7%, *n* = 59). Other reasons included being provoked by the “devil” or “bad spirits” (9.9%, *n* = 35), sadness or depression (7.4%, *n* = 26), because it is “fashionable” (7.1%, *n* = 25), and dual use with *khat* (5.1%, *n =* 18). On the other hand, when asked about the reasons why someone might start using smokeless tobacco products, more than half of the respondents reported: “for medicinal purposes” (50.7%, *n* = 179). The next most common response was “don’t know” (24.6%, *n* = 87). Other reasons included: to “protect from evil spirits” (10.8%, *n* = 38); “experimentation” (8.5%, *n* = 30); “to keep warm” or “fight the wind” [i.e., keep warm] (8.5%, *n* = 30); “provoked by the devil” or “lack of religion” (7.6%, *n* = 27); the influence of older relatives (6.5%, *n* = 23); and peer pressure (5.9%, *n* = 21).

Respondents also reported a number of reasons other than for personal consumption why someone might be motivated to grow or use tobacco; the most common being “to sell” (83.6%, *n* = 295), followed by “to repel snakes” (35.4%, *n* = 125), “for medicinal use with animals” (26.3%, *n* = 93), and “to kill leeches” (9.9%, *n* = 35).

### Exposure to pro- and anti-tobacco messaging

More than 45% percent of the respondents reported having seen point-of-sale advertising for cigarettes within the last 30 days (Table 3). Residents living in the town were more likely to have encountered this form of pro-tobacco messaging than respondents residing in the outlying rural areas (*p* = .055). However, overall, more respondents (70.3%) reported having been exposed to one or more forms of anti-tobacco messaging within the last 30 days than to pro-tobacco advertising (49%) (Table 3). There were significant differences in respondents’ reports based on place of residence in relation to having seen or heard anti-tobacco messaging on television (47.7% among those residing in the town vs. 20.7% among those residing in an outlying rural *kebele*, *p* < .001) and in receiving anti-tobacco messaging from health extension workers (28.5% among those in a rural *kebele* vs. 16.1% among those residing in town, *p* = .007).

**Table 3:** Exposure to pro- and anti-tobacco messaging

*N* = 353		
Variable	*n*	%
Seen/heard advertising *for* cigarettes, in last 30 days, in ≥1 locations	173	(49.0)
In stores where cigarettes are sold	162	(45.9)
Radio	25	(7.1)
Public transportation	19	(5.4)
Television	12	(3.4)
Seen/heard messaging *against tobacco* use, in last 30 days, in ≥1 of following locations:	248	(70.3)
Radio	203	(57.5)
Television	120	(34.0)
Health Extension worker	79	(22.4)
Public social gatherings	35	(9.9)
Newspapers or magazines	33	(9.3)
Church/Bible^a^	32	(9.1)
School^a^	12	(3.4)

^a^Category elicited by asking “Anywhere else?”

## Discussion

This study is the first to report on KABs related to maternal tobacco use and SHS exposure, *khat* use, the role of religion in tobacco-related attitudes, non-personal uses of tobacco, and level of exposure to pro- and anti-tobacco messages among women in Ethiopia. While general awareness of harm associated with tobacco use was high, specific knowledge of associated health risks was minimal. Respondents reported low levels of social acceptability of female tobacco use. However, a majority were able to name personal and non-personal benefits of using tobacco products. Overall, the respondents reported more frequent exposure to anti-tobacco messaging versus pro-tobacco messaging, in the last 30 days.

While respondents’ general awareness of harm associated with tobacco use was high, awareness of specific diseases or health conditions caused by maternal smoking of cigarettes and exposure to SHS was limited. This lack of awareness of specific conditions is consistent with findings from studies that have explored KABs in other LMICs [31, 37–45]. Overall, these findings lend support for tobacco control efforts aimed at increasing awareness of specific health risks associated with tobacco use and SHS exposure, particularly concerning the risks of addiction. Additionally, follow-up studies are needed to further explore harm perceptions associated with SHS, particularly in relation to children and pregnancy, as well as the actions that women and family members may currently be taking to protect themselves and their children from exposure to SHS.

In the described setting, the barrier of social acceptability appears to be intact, with more than 96% of the respondents reporting that it was unacceptable for women to smoke, use smokeless tobacco products, or chew *khat*. Based on available data from LMICs, there is significant national and regional variability in relation to women and men’s attitudes towards women's use of tobacco [23, 24, 31, 39, 43, 45]. It has, however, been observed that when women’s status and level of empowerment in a country rises, a reciprocal rise in the prevalence of tobacco use is often observed [1, 4]. In the past, tobacco control efforts have tended to reinforce social and cultural norms that inhibit the uptake of smoking by girls and women. However, this has also resulted in a number of unintended negative consequences, including stigmatization of smokers rather than the acts of smoking. In response, some authors have begun to advocate for tobacco control policies and programs that are simultaneously committed to preventing women’s tobacco use and ensuring that these efforts are also contributing to, and not impeding, improvements in women’s socio-economic status [46–50]. Sub-Saharan Africa may benefit from the lessons learned and join forces with other sectors (e.g., human rights, economic development) in addressing tobacco control as a social justice issue.

All respondents reported having a religious affiliation, and among the subsample, all reported that they perceived tobacco as “bad” and that their religion told them that tobacco was “bad.” The influence of religion on tobacco-related behaviors represents a socio-cultural dynamic at play in this setting. In large representative samples in the United States studies have continued to find associations between lower smoking prevalence and higher frequency of attendance at religious services [51, 52]. Other studies using large nationally representative databases and prospective designs have continued to find similar associations; however, mediating factors are not well understood [52, 53]. In this study, while a majority reported that their religion had significantly influenced their attitude toward tobacco use, no significant differences were found based on religious affiliation. However, the open-ended responses provided for why someone might start using tobacco suggest that respondents adhered to both traditional beliefs about tobacco use and dominant religious ideology (i.e., Protestant, Orthodox Christian, and Muslim). On the one hand, being “provoked by a devil or bad spirit” was offered as a reason for starting to use either cigarettes (*n* = 35) or smokeless tobacco (*n* = 27), yet a similar number (*n* = 38) reported a reason to start using smokeless tobacco would be to “protect from evil spirits”. Due to the lack of variability in the data, no associations can be identified between smoking and religious affiliation. However, the findings suggest that religion may have a protective effect. Therefore, to understand the potential role of religion in the prevention of the uptake of tobacco use in Ethiopia further exploration of differences by religious affiliation, and level of religiosity or commitment, is warranted [54].

Despite the low prevalence of cigarette smoking and smokeless tobacco use, participants in this study were able to provide a range of reasons why someone might start using these products. Notably, more than half of the respondents named “medicinal purposes” as a reason for initiating smokeless tobacco use. Similar observations have been documented in other studies conducted among women in LMICs [37, 38, 44, 55]. In these studies, the reported indications for using various forms of smokeless tobacco have ranged from treating pregnancy-induced nausea, to suppressing appetite and treating headaches. Further research is needed to understand the specific conditions for which individuals in this setting use smokeless tobacco.

A number of reasons why someone might grow or use tobacco (other than for personal consumption) were identified. More than 83% indicated it was a crop that may be grown for income; however, less than 6% reported that a member of their household was currently involved in the growing, manufacturing or selling tobacco. When queried on how tobacco is used to repel snakes, explanations were given indicating that tobacco was both planted around the exterior of the home and the juice from chewing tobacco was used to spit directly onto the snake. Respondents also reported that tobacco leaves are used to make a powdered “medicinal” product for their livestock to treat ingested leeches and to kill leeches by adding it to sources of drinking water. Traditional uses of tobacco have been studied in other settings, and the role that this cultural-environmental factor plays in perpetuating a perceived need for tobacco in the community should be explored further and taken into consideration when developing contextualized tobacco control interventions in rural Ethiopia [56].

Studies conducted among women in LMICs have demonstrated that exposure to cigarette advertising is positively associated with more favorable attitudes toward smoking [23, 24]. In this study, nearly half of the respondents reported having seen point-of-sale advertising within the last 30 days. In contrast, reports of exposure to other common locations for tobacco advertising were negligible. The fact that tobacco advertising has been curtailed is a noted strength in Ethiopia's current tobacco control context, as the enforcement of bans on tobacco advertising is one of the WHO FCTC’s evidence-based recommended tobacco control strategies [57]. The current situation in Ethiopia may be unique compared to other countries where pro-tobacco messages are ubiquitous. However, it should be noted that while no significant differences were observed in respondent’s tobacco-related KABs based on place of residence, even this small degree of difference in urbanicity (residing in a rural town vs. outlying rural area) resulted in increased exposure to tobacco advertising, making a case for increased monitoring in rural towns and emerging urban areas. While tobacco advertising is currently prohibited in Ethiopia; resources to monitor enforcement remain limited but should be prioritized [58].

Of particular interest, respondents reported a greater frequency of exposure to anti-tobacco messaging than to pro-tobacco messaging (Table 3). These findings provide evidence of the current reach of anti-tobacco messaging on the radio (57%) and on television (34%). Further research would be needed to understand the nature and sources of these messages, including the effectiveness of the current government-sponsored public awareness campaigns. Respondents also reported exposure to a number of other sources of anti-tobacco messaging that could be described as more proximal in origin—i.e., from the local social and built environment. These sources included health extension workers, public social gatherings, churches, and schools. This current dynamic represents a community strength that could be reinforced by continuing to involve local entities in public awareness campaigns, and by exploring the possibility of accessing these networks to disseminate interventions. Additionally, based on research from other LMICs, these conditions suggest that basic awareness measures (including surveillance activities) could lead to significant increases in adoption of household smoking bans, especially among households with no smokers [59]. In turn, higher adoption of smoking bans would also have the potential to influence community norms related to tobacco use and SHS exposure.

### Strengths and limitations

Strengths of this study include the use of items from standardized, validated instruments and the use of standardized translation and adaptation guidelines used previously in LMICs [31, 34, 60, 61]. The exploratory nature of this study provides baseline data on this issue. However, the sample size may have limited the ability to detect statistically significant associations between outcome variables and covariates. Furthermore, while utilization of a systematic sampling technique to select households enhanced the generalizability of the findings for women in this region, due to the restrictive inclusion criteria and sample frame the findings cannot be generalized to women across Ethiopia. Additionally, it is noted that because the survey was conducted during the day and 20% (*n* = 137) of the households approached were not at home, this may have introduced selection bias, as it is unknown whether these residents may have differed from those that were home during the day. Finally, while the use of an interviewer-administered survey was useful in addressing potential literacy barriers, it may also have introduced social acceptability bias, especially in settings with high levels of tobacco-related stigma and prescribed gender roles [35].

## Conclusions

This study contributes to the current understanding of tobacco-related knowledge, attitudes, and beliefs held by Ethiopian women in the Aleta Wondo region. The findings from this study can help to inform the development of contextualized gender-specific primary prevention tobacco control interventions, particularly with the promotion of smoke-free homes. There appears to be a window of opportunity to build on the current level of awareness of health risks associated with tobacco and limited exposure to pro-tobacco message to proactively prevent the uptake of tobacco use by both women and men and to prevent the normalization of smoking indoors and in public spaces. Implementation of the recently enacted comprehensive tobacco control measures is urgently needed, as is the promotion of smoke-free homes. Targeted educational campaigns that increase awareness of specific risks associated with tobacco use, SHS exposure and addiction may counteract the perceived benefits related to smoking. Additionally, when planning interventions and future research, we highlight the need to consider the unique cultural-environmental contextual factors, including traditional and non-personal uses for tobacco, which may perpetuate the perceived need for tobacco. Finally, community strengths should be built upon by reinforcing and expanding the role that local community entities, including faith communities, play in the ongoing dissemination of anti-tobacco messaging.

## Abbreviations

EDHS: Ethiopian Demograghic Health Survey
KABs: Knowledge, attitudes and beliefs
LMICs: Low- and middle-income countries
MPI: Multidimensional Poverty Index
SHS: Secondhand smoke
WHO FCTC: World Health Organization Framework Convention on Tobacco Control
